# Gemcitabine improves survival in patients with recurrent or metastatic nasopharyngeal carcinoma

**DOI:** 10.1186/s40880-016-0163-6

**Published:** 2016-12-22

**Authors:** Shaodong Hong, Li Zhang

**Affiliations:** 1 Department of Medical Oncology, State Key Laboratory of Oncology in South China, Collaborative Innovation Center for Cancer Medicine, Sun Yat-sen University Cancer Center, Guangzhou, 510060 Guangdong P. R. China; 2 Department of Medical Oncology, Sun Yat-sen University Cancer Cente, 651 Dongfeng Road East, Guangzhou, 510060 Guangdong P. R. China

**Keywords:** Metastasis, Nasopharyngeal carcinoma, Chemotherapy

## Abstract

For decades, the selection of chemotherapeutic regimens for the treatment of recurrent or metastatic nasopharyngeal carcinoma has been mainly empirical. To our knowledge, there is no phase 3 trial that has been conducted to determine the optimal treatment for these patients before our publication. Recently, we published an article in *The Lancet* entitled “Gemcitabine plus cisplatin versus fluorouracil plus cisplatin in recurrent or metastatic nasopharyngeal carcinoma: a multicentre, randomised, open-label, phase 3 trial.” The results of our study indicate that gemcitabine plus cisplatin could improve the survival of patients with recurrent or metastatic nasopharyngeal carcinoma compared with conventional fluorouracil plus cisplatin.

## Background

Nasopharyngeal carcinoma (NPC) is an endemic cancer, with the highest incidence in Southeast Asia [1, 2]. Radiotherapy or chemoradiotherapy has become the primary treatment of early or locoregionally advanced NPC [3, 4]. However, about one-third of these patients have treatment failure due to distant metastasis [5]. The median overall survival (OS) of recurrent or metastatic NPC patients is only approximately 20 months [6]. Fluorouracil plus cisplatin is generally regarded as the standard first-line chemotherapy regimen for these patients, although it has never been directly compared with best supportive care. However, the fluorouracil regimen is limited by the requirement for deep-vein catheterization and the short duration of response. Therefore, using carefully designed, large clinical trials to find novel agents for the treatment of metastatic NPC is of critical importance. As such, in our recent *Lancet* article entitled “Gemcitabine plus cisplatin versus fluorouracil plus cisplatin in recurrent or metastatic nasopharyngeal carcinoma: a multicentre, randomised, open-label, phase 3 trial,” [7] we presented the preliminary results of our study of the efficacy and safety of two competing cisplatin-based combinations—gemcitabine plus cisplatin versus fluorouracil plus cisplatin—in patients with recurrent or metastatic NPC.

The study was conducted in 22 centers in China. In these centers, Epstein-Barr virus is the major etiology of NPC. A total of 362 patients were randomized in a 1:1 ratio to receive either gemcitabine plus cisplatin or fluorouracil plus cisplatin for a maximum of six 21-day treatment cycles. This was an open-labelled study. The primary endpoint was progression-free survival (PFS). The key findings were the statistically and clinically significant survival improvement of patients who received gemcitabine plus cisplatin compared with patients who received fluorouracil plus cisplatin, in both PFS [median: 7.0 vs. 5.6 months; hazard ratio (HR) 0.55; *P* < 0.001] and OS (median 29.1 vs. 20.9 months; HR 0.62; *P* = 0.003). Although the follow-up time for OS was relatively short, the substantial gain in OS might be explained by the low crossover rate of 8% to gemcitabine in the fluorouracil-plus-cisplatin group. Final OS results with more in-depth analyses are being generated.

Our exploratory analyses further suggest that the improvement of PFS for patients who received gemcitabine plus cisplatin was consistent across most subgroups. However, it should be noted that the enrolled patients were from endemic areas, where the primary NPC histological classifications are non-keratinizing undifferentiated (type III) and non-keratinizing differentiated (type II) diseases. For keratinizing subtype (type I), which is more prevalent in Western countries, whether gemcitabine is superior to fluorouracil needs more investigation. Overall, gemcitabine demonstrated more remarkable anti-tumor activity compared with fluorouracil, as seen by the significant improvement in objective response rate (64% vs. 42%, *P* < 0.001). Disease control rates were similarly high in both arms (90% vs. 86%).

The adverse events of both regimens were as expected. Patients who received gemcitabine had increased risks of grade ≥3 leukopenia, neutropenia, and thrombocytopenia, whereas those who received fluorouracil had an increased risk of grade ≥3 mucosal inflammation. The occurrence rates of serious adverse events were similar between the two arms (4% in the gemcitabine-plus-cisplatin group vs. 6% in the fluorouracil-plus-cisplatin group).

The next frontier in the treatment of NPC patients will entail improved prognosis stratification with existing and novel biomarkers and the development of novel drugs beyond conventional chemotherapeutics [8].

## Conclusions

Our data indicate that, for patients with recurrent or metastatic NPC, gemcitabine is superior to fluorouracil in terms of OS and PFS. These results could establish gemcitabine plus cisplatin as the new standard first-line treatment regimen for this patient population.
